# Alternative approaches to target Myc for cancer treatment

**DOI:** 10.1038/s41392-021-00500-y

**Published:** 2021-03-10

**Authors:** Chen Wang, Jiawei Zhang, Jie Yin, Yichao Gan, Senlin Xu, Ying Gu, Wendong Huang

**Affiliations:** 1 Division of Medical Genomics and Cancer Institute (Key Laboratory of Cancer Prevention and Intervention, Ministry of Education), the Second Affiliated Hospital, School of Medicine, Zhejiang University, Hangzhou, China; 2 Institute of Genetics, Zhejiang University and Department of Genetics, School of Medicine, Zhejiang University, Hangzhou, China; 3 Zhejiang Provincial Key Lab of Genetic and Developmental Disorder, Hangzhou, Zhejiang 310058 China; 4 Molecular and Cellular Biology of Cancer Program & Department of Diabetes Complications and Metabolism, Beckman Research Institute, City of Hope, Duarte, CA USA; 5 Irell & Manella Graduate School of Biological Sciences, Beckman Research Institute, City of Hope, Duarte, CA USA; 6 Zhejiang Laboratory for Systems & Precision Medicine, Zhejiang University Medical Center, Hangzhou, 311121 China

**Keywords:** Cancer, Oncogenes

## Abstract

The Myc proto-oncogene family consists of three members, *C-MYC*, *MYCN*, and *MYCL*, which encodes the transcription factor c-Myc (hereafter Myc), N-Myc, and L-Myc, respectively. Myc protein orchestrates diverse physiological processes, including cell proliferation, differentiation, survival, and apoptosis. Myc modulates about 15% of the global transcriptome, and its deregulation rewires the cellular signaling modules inside tumor cells, thereby acquiring selective advantages. The deregulation of Myc occurs in >70% of human cancers, and is related to poor prognosis; hence, hyperactivated Myc oncoprotein has been proposed as an ideal drug target for decades. Nevertheless, no specific drug is currently available to directly target Myc, mainly because of its “undruggable” properties: lack of enzymatic pocket for conventional small molecules to bind; inaccessibility for antibody due to the predominant nucleus localization of Myc. Although the topic of targeting Myc has actively been reviewed in the past decades, exciting new progresses in this field keep emerging. In this review, after a comprehensive summarization of valuable sources for potential druggable targets of Myc-driven cancer, we also peer into the promising future of utilizing macropinocytosis to deliver peptides like Omomyc or antibody agents to intracellular compartment for cancer treatment.

## Introduction

**Fig. 1:**
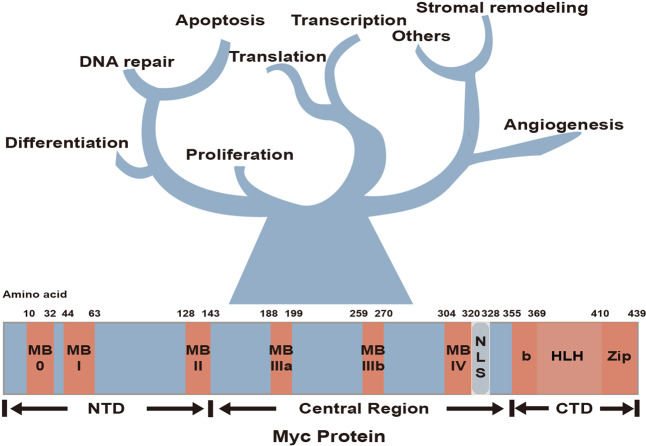
Structure of Myc protein and its fundamental contributions to diverse biological processes. Myc is comprised of three domains: NTD, central region, and CTD. Highly conserved elements, Myc boxes, are staggered across these domains and involved in the functions of Myc. MBI and MBII carry out the transactivation of Myc target genes, while MB III regulates the stability of Myc. The BR/HLH/LZ motif at CTD is related to the heterodimerization with Max and DNA–protein interaction. The physiological transcription factor Myc thrives diverse fundamental biological processes, including proliferation, differentiation, transcription, translation, apoptosis, angiogenesis, DNA repair, and stromal remodeling

The *MYC* proto-oncogene was originally identified as the cellular homolog to the viral oncogene (*v-myc*) of the avian myelocytomatosis retrovirus.^1^ *MYC* gene and its paralogs (*MYCN* and *MYCL*) encode the transcription factors that engage in modulating over 15% of global transcriptome from flies to humans.^2^ Myc protein has three critical domains: (1) the amino-terminal domain (NTD) harbors conserved Myc boxes I and II (MBI and MBII), which are essential for the transactivation of Myc target genes; (2) the basic (b) helix-loop-helix (HLH) DNA binding domain; (3) the carboxy-terminal domain (CTD), which comprises Myc leucine zipper domain (Fig. 1). The functionally diverse repertoire of genes controlled by Myc participate in a broad spectrum of intracellular biological processes, including proliferation, differentiation, apoptosis, DNA damage repair, metabolism, and extracellular biological events such as angiogenesis and stromal remodeling^2–5^ (Fig. 1).

**Fig. 2:**
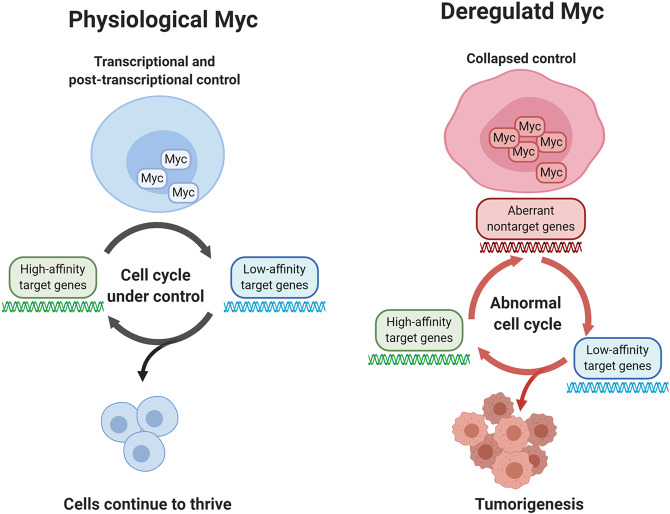
Comparison between physiological Myc and deregulated Myc. The collapsed control of Myc intensifies the expression of both target genes as well as additional aberrant nontarget genes, thereby enhancing tumorigenesis. In the physiological state, Myc is tightly controlled by transcriptional as well as post-transcriptional components. High-affinity target genes, with expression independent of the Myc level and cell types, actively respond to Myc, while low-affinity target genes respond at a low level. The integrated signals from these two target genes facilitate the cells to thrive. Decontrolled Myc increases the expression of both high- and low- affinity target genes; also, additional nontarget genes might be activated. These three signals lead to further tumorigenesis

Under normal circumstances, Myc protein, as well as its familial members, is under stringent control by multiple mechanisms; however, Myc is frequently dysregulated during tumorigenesis. The uncontrolled Myc is a detrimental genomic dominator that intensifies the expression of both target genes as well as aberrant nontarget genes to promote tumorigenesis (Fig. 2). As expected, the *MYC* oncogene is a central driver in multiple cancers, such as breast cancer,^6^ liver tumor,^7^ colorectal carcinoma,^8^ and prostatic neoplasia.^9^ High and/or aberrant Myc expression occurs in >70% human cancers and is related to poor prognosis and aggressive conditions.^10–12^ Thus, the high recurrence of Myc overexpression in cancers and its universal role in transcriptional regulation deems it as a tricky oncoprotein for targeting.

Due to the dual pivotal roles in normal cells and cancerous cells, targeting Myc oncoprotein is a rather challenging but urgent requirement. Traditional chemotherapy has contributed enormously to current medicine development and plays an essential role in the treatment of cancers with overexpressed Myc. The traditional no-discrimination chemotherapy not only kills the cancer cells but also undermines the growth of normal proliferating cells. This method is gradually fading while precise and effective targeted therapeutics are increasing. Patient-friendly and effective drugs, such as Gleevec, with specific antitumor activity, have changed the paradigm of cancer treatment. The success of Gleevec in the treatment of chronic myeloid leukemia (CML) indicates the importance of discriminating against the diverse genomic or molecular characters in different cancers. Thus, the current consensus has emerged as targeting critical nodes responsible for cancer development and maintenance to achieve a selective antitumor effect. The phrase “oncogene addiction” was first coined to describe the necessity of continuing activity of oncogenes for cancer maintenance. And oncogenes, whose inhibition leads to tumor cell death and reduced tumor burden, have been of great interest as therapeutic targets. Interestingly, the genetic inhibition of tumor-preferred oncogene *MYC* relieves the tumor burden. As illustrated by mouse models with an inducible *MYC*, Myc-driven skin papillomas and osteosarcomas can be reversed upon *MYC* deactivation.^13,14^ Since *MYC* is a potent driver gene in multiple human cancers, this oncoprotein has been listed on the top of the putative drug target list.

Despite the long pursuit of inhibitors for Myc, there is a dearth of information, and directly targeting the Myc oncoprotein is yet challenging. Briefly, two major problems need to be considered. First, Myc acts as a general transcription factor for both normal proliferating cells and tumor cells. Myc transcription factor is ubiquitously expressed and activated during normal embryogenesis and in the tissue compartments with high proliferative capacity such as skin epidermis and gut.^12^ Furthermore, the expression of Myc protein is strongly correlated with cell proliferation. Although the inhibition of Myc induces tumor regression in mouse models, the dreadful outcomes of directly targeting the Myc transcription factor through genetic manipulation of Myc or directly destroying Myc oncoprotein were daunting for a long period. However, some recent studies relieved these reasonable concerns about the catastrophe outcomes of partial depletion or inhibition of Myc. As reported, *MYC* haplotype mice showed no-phenotype during normal development and even enhanced longevity.^15^ Also, a systemic Myc inhibition administered to a preclinical mouse model of Ras-induced lung adenocarcinoma showed well-tolerated effects on normal regenerating tissues.^16^

Second, the structure of the Myc protein itself does not have preferred binding pocket for traditional drug modalities. Myc protein lacks the catalytic clefts, and thus, has no feasible or effective drug-binding pockets for traditional small molecule drugs. Moreover, its predominant nucleus localization is inaccessible to antibody-based agents. Hence, alternative approaches need to be explored to bypass these obstacles.

With respect to alternate targeting of Myc in Myc-dependent neoplasms, approaches to treat Myc-deregulated cancer are mostly fall into five catagories: (1) targeting *MYC* transcription; (2) targeting *MYC* mRNA translation; (3) targeting Myc stability; (4) targeting Myc–Max interaction; (5) targeting accessibility of Myc to downstream genes. However, multiple alterations in cancer might generate phenotypes akin to Myc deregulation. The gross genetic changes at *MYC* locus, including insertional mutagenesis, chromosomal translocation, and gene amplification would lead to the phenotype of Myc deregulation in tumor. Besides, abnormal activation of the intracellular signal network could converge on Myc expression or protein stabilization and confer Myc deregulated phenotype to cells. For example, Ras activation could lead to Myc deregulation. Perhaps directly targeting such a high hierarchical component in signal pathway may not successfully inhibit Myc overexpression as cancer cells generally have cross-talking in upstream signal pathways and might circumvent these upstream components to maintain the state of Myc deregulation. Indeed, deregulated Myc induced by these alterations incurs cellular stress, such as endoplasmic reticulum (ER) strss, replication strss, and mRNA-splicing stress. Accordingly, cells harbored deregulated Myc would rewire signal pathways and adapt to such circumstance. Hence, cancer cells could be selectively killed through targeting critical nodes in these rewired pathways. And this, actually, is consistent with the concept of synthetic lethality.

Currently, the genetics or epigenetics signatures identified from “omic” datasets provide valuable resources for searching alternative targets of the Myc oncoprotein. Discovering the vulnerable and druggable nodes underlying Myc deregulation is pivotal for the alternative targeting of Myc for cancer treatment. A myriad of studies has recently identified suitable candidates to alternatively target Myc. In this review, we summarized the latest advances with respect to Myc targeting and provide a basis for future investigation. Also, we peer into the promising future to target Myc from the preclinical success of Omomyc.

## Alternative methods to target myc

**Fig. 3:**
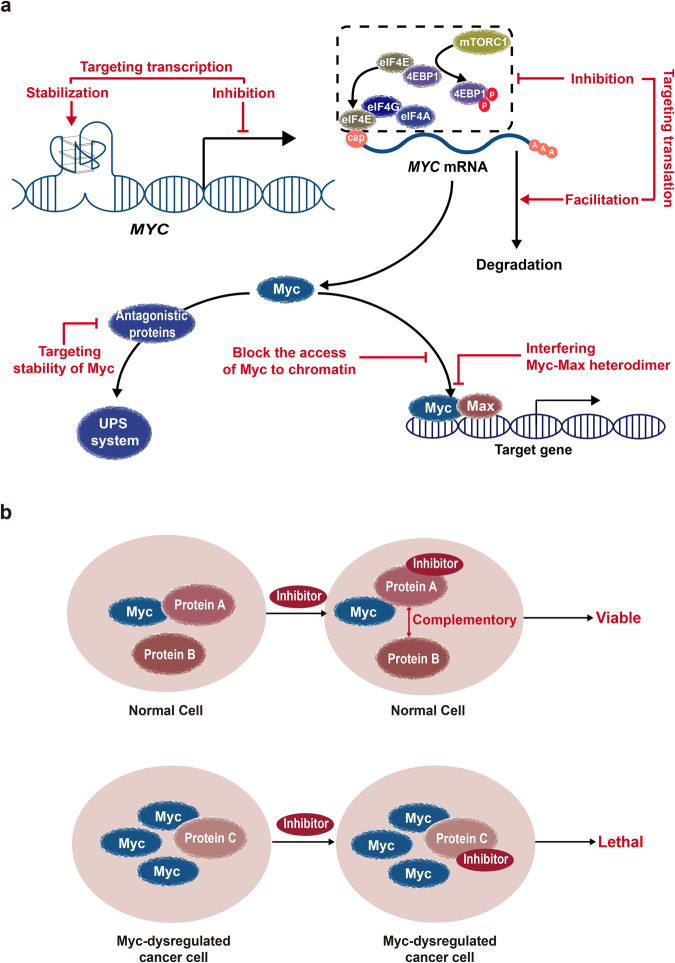
Alternative approaches to target Myc. **a** Targeting Myc through the downregulation of Myc protein level and restraining the oncogenic activity of hyperactivated Myc. Targeting the transcription of Myc is a straightforward method to target Myc alternatively either through stabilizing the G-quadruplex structure with small molecules or blockading the signal transduction from *MYC* to RNA polymerase. The inhibition of the translation process from *MYC* mRNA to Myc through targeting critical proteins involved in the translation initiation or *MYC* mRNA stability could also be considered. Other alternative ways to target Myc are hijacking the UPS system to downregulate the Myc protein level, blocking the free access of Myc to its target, and disrupting the interface between Myc and Max. **b** Targeting Myc through synthetic lethality. Myc-deregulated cancer cells reshuffle the inner signal modules and rely on specific signal components to meet the growing demands to thrive as compared to the normal cells. The inhibition of such components leads to synthetic lethality of cancer cells with Myc overexpression. Conversely, normal cells have abundant complementary signaling pathways to buffer such inhibition and yet be viable

Given the lack of favorable chemotherapeutic drugs for Myc-driven cancers and the dilemmas of targeting Myc oncoprotein directly, alternative methods have been forged to target Myc. This process is categorized as follows: downregulating the level of Myc oncoprotein either at the transcriptional or post-transcriptional level, restraining the oncogenic activity of hyperactivated Myc either through disassociating the Myc–Max interaction or blocking the free access of Myc to downstream genes, and targeting the vulnerability of Myc-dependent tumor cells through efficient synthetic lethal combination (Fig. 3). For instance, the inhibition of the initiation of *MYC* transcription via targeting BRD4 or stabilizing the structure of G-quadruplexes provides an in-depth insight into the cancer treatment. In addition, the inhibition of *MYC* mRNA translation by targeting proteins, such as 4EBP1, is also a promising alternative. Furthermore, the destabilization of Myc could be handy for the treatment of Myc deregulated cancer.^17,18^ Also, restraining the oncogenic activity of Myc through disassociation of Myc–Max heterodimer and abrogating the free access of Myc to downstream genes is another approach to alternative Myc targeting.

Certainly, the concept of selective killing cancer cells and leaving normal cells nearly untouched is figuring prominently. As mentioned above, oncogenic Myc remodules signal nodes to support its tumorigenesis and maintenance. Therefore, target these rewired nodes through solid synthetic lethal interaction may shed light on targeting undruggable proteins like Myc. Briefly, synthetic lethal interactions are the specific vulnerability of cancer cells as compared to normal cells. The process of tumorigenesis would re-deploy its signal modules, and thus confers the selective growth advantage to cancer cells. To some extent, such advantage is also a vulnerability when we decide to target cancers through synthetic lethality, for example, utilizing poly (ADP)-ribose polymerase (PARP) inhibitors to treat *BRCA1* and *BRCA2* mutant breast cancers. Given the promising future of synthetic lethality in targeting “undruggable” oncoproteins, surfacing the reliable synthetic lethal interactions becomes pivotal to target “undruggable” oncoproteins, such as Myc and further translate it into clinical practice. As the high-throughput deep-screening technology continues to thrive, synthetic lethality screening progresses gradually. Over a hundred genes have been discovered for their potential synthetic lethality with Myc. Importantly, synthetic lethality performs exceeding its inherent favorable selectivity. The success of synthetic lethal clinical practice with PARP inhibitors in *BRCA1* and *BRCA2* mutant breast cancers shapes the bright future for selective and efficient cancer treatments based on molecular features. Therefore, oncoproteins hold enormous promise for future cancer treatment, especially those that are undruggable, such as Myc and Ras, but critical for tumorigeneses.

### Targeting *MYC* gene transcription

The inhibition of Myc transcription falls into two categories: first, structure-oriented methods that stabilize the G-quadruplex structure with small molecules; second, targeting the corresponding proteins involved in the *MYC* transcription complex. G-quadruplexes are noncanonical DNA structures located adjacent to the promoter regions of oncogenes, such as *MYC*,^19^ and its three-dimensional structure offers natural binding pockets for small molecules, which might, in turn, downregulate the expression of Myc through small molecule intervention. The G-quadruplexes regulate the gene expression, including 90% of *MYC* expression,^19^ thus making it a potential drug target for Myc-deregulated cancer. Besides, the chromatin-dependent signal transduction from *MYC* to RNA polymerase exhibits some specific nodes for Myc targeting. Myc transcription is relevant to the acetylation of histone lysine, which is associated with transcriptional activation. Histone acetylation is necessary for recruiting acetyl-lysine binding modules or bromodomains for further organization of the exquisitely arranged transcriptional complexes.^20^

#### Stabilization of G-quadruplexes with small molecules

Guanine-rich DNA sequences can fold into four-stranded, noncanonical, secondary structures known as G-quadruplexes,^21^ which are characterized by stacks of Hoogsteen-bonded guanine tetrads stabilized by central potassium ions and flanked by loop regions.^19^ Furthermore, the G-quadruplexes are involved in essential genome functions, such as transcription, replication, genome stability, and epigenetic regulation, together with numerous connections to cancer biology.^21^ The quadruplex motifs are frequently over-represented in genes, which promote tumorigenesis.^21^ Interestingly, the experimental data have shown that the G-quadruplex structures are frequently present in cancer states than in normal states of the cells.^21^ For example, higher levels of G-quadruplex structures were observed in antibody staining of stomach and liver cancer tissues as compared to their normal counterparts. Intriguingly, *MYC* is a robust driver gene in human cancers, which has a G-quadruplex DNA motif in the proximal G/C-rich promoter region, known as the nuclease hypersensitive element III1 (NHE III1; also known as the CT element). Hence, finding suitable small-weight molecules with high affinity and selectivity to the G-quadruplex of Myc and predictable actions inside the cell compartment is essential. QN-1, a difluoro-substituted quinoxaline, is recently reported to have selective and potent binding to the G-quadruplex of Myc. QN-1 has a significant *MYC* binding preference among genes with G-quadruplexes, including *VEGF*, *BCL2*, and *HRAS*. Besides, QN-1 potently downregulates *MYC* transcription in triple-negative breast cancer (TNBC) cells and effectively suppresses tumor growth in a TNBC mouse model.^22^ One of the most cutting-edge researches related to small-molecule stabilizers of G-quadruplexes in handling Myc-deregulation is the Phase Ib study of APTO-253 in patients with relapsed or refractory acute myelogenous leukemia or high-risk myelodysplasia.^23^ This inspiring clinical trial showed well-tolerated outcomes at the recommended dose in Phase 2 and evidence of antitumor activity in the form of stable disease in patients with advanced solid tumors. Nevertheless, small molecules, which bind DNA, may still raise reasonable concerns about multiple mechanisms of action and genome-wide catastrophe. Recently, *DDX5*, which encodes the DEAD-box protein 5 (DDX5), also named p68, is involved in unfolding the G-quadruplex of Myc.^24^ DDX5 belongs to the DEAD-box RNA helicase family, and DEAD-box (DDX) proteins form the largest family of double-stranded RNA (dsRNA) helicases. It contain 12 conserved sequence motifs, including the characteristic Asp–Glu–Ala–Asp (DEAD) motif. Typically, the protein level of DDX5 is low in normal cells, but is frequently overexpressed in a broad range of tricky human cancers such as colon, lung, breast, and prostate cancers. Also, DDX5 is implicated to promote tumorigenesis, tumor progression, and cellular transformation. Importantly, it stimulates the expression of *MYC* in multiple solid tumors and executes the efficient unfolding of the G-quadruplex of Myc, which is a poised structure for gene transcription. The pharmacological inhibition of DDX5 may have a favorable effect on cancer cells, especially those harbor high protein level of DDX5 and Myc.

#### Interfering the signal transduction from MYC to RNA polymerase

Mammalian bromodomain and extraterminal (BET) proteins facilitate transcription by assembling the scaffolds with other co-regulatory proteins, including mediator subunits and the positive transcription elongation factor-b (PTEF-b) complex at enhancers.^25^ BRD4 is a member of the BET family and a critical epigenetic regulator. Specifically, BRD4 participates in transcription by recruiting PTEF-b to the appropriate site of hyperacetylated chromatin, followed by phosphorylation of the carboxy-terminal domain of RNA polymerase II. Consequently, RNA polymerase II is free from the promoter-proximal region and ready to initiate transcriptional elongation. A selective orally available BET/BRD4 bromodomain inhibitor, AZD5153, could displace BRD4 from chromatin.^26^ Displacement of BRD4 from the super-enhancers within the *MYC* oncogene leads to a dose-dependent decrease in Myc mRNA and protein levels in both cancer cells and animal models of hematological malignancies. Another BET inhibitor, GSK525762, has entered the Phase 2 stage with manageable and reversible adverse events.^27^ It significantly reduces the Myc expression in both prostate cancer cell lines and a patient-derived tumor model with high Myc expression.^28^ Thus, BET inhibitors, like AZD5153 and GSK525762, could be promising candidates for further clinical usage in Myc-deregulated cancer.^29^

Besides, a strategy or technology termed as proteolysis targeting chimera (PROTAC) has been developed recently to hijack the Cereblon E3 ubiquitin ligase for degrading specific oncoproteins, such as BRD4, potently and constantly.^30^ PROTAC was first used as a chemical knock-down approach with reversibility and has been recently explored to satisfy additional needs, including drug discovery.^30^ This could be a potent method to degrade target proteins, especially those that have a long half-life in the physiological circumstances, and supplement new approaches to alternatively target Myc oncoprotein. Actually, a new BRD4-specific inhibitor based on the strategy of PROTAC has been developed by conjugating the small molecule BET inhibitor JQ1 with a phthalimide moiety. This inhibitor, dBET, demonstrated the similar selectivity towards the BET family of bromodomains as JQ1.^31^ dBET1 is a specific protein degrader that results in a manipulated, persistent, and significant loss of BRD4 protein, as tested in a human AML cell line, MV4-11. Furthermore, BRD4 degradation induced by dBET1 results in the downregulation of *MYC* transcription and antitumor activity in human leukemia xenograft.^31^ Thus, BET inhibitors might contribute to the clinical utility for treating Myc-deregulated cancers, and, sometimes, combination other agents with BET inhibitors may have more potent effect as BET inhibitors alone may lead to drug resistance.^32^

### Targeting *MYC* mRNA translation

Cells have developed extraordinary mechanisms to adequately control and supervise the overall protein expression and mRNA translation. In mammals, the mammalian target of rapamycin complex 1 (mTORC1) is the key player of protein synthesis. *MYC* mRNA translation is orchestrated by diverse proteins, including mTORC1 and its pivotal downstream effectors: p70S6Kinase 1 (S6K1) and eukaryotic translation initiation factor 4E (eIF4E) binding protein 1 (4EBP1). S6K1 is directly phosphorylated by mTORC1, and in turn, it phosphorylates substrates that propel further translation initiation. 4EBP1 blocks the binding of eukaryotic translation initiation factor 4G (eIF4G) to eIF4E and hinders mRNA translation, which is compromised when mTORC1 phosphorylates 4EBP1. The mRNA complex structures also influence the successful translation. The secondary structure of the 5′-untranslated region (UTR) of *MYC* mRNA forms G-quadruplexes, which further hinder the initiation of *MYC* mRNA translation. Eukaryotic translation initiation factor 4A (eIF4A) unwinds the G-quadruplexes during scanning as a prelude to its association with the 43S preinitiation complex.^33,34^ The helicase activity of eIF4A is enhanced by eIF4B, a downstream effector of S6K1. Also, eIF4A is a crucial member of the active eIF4F complex, and the latter is associated with poor prognosis and drug resistance in various cancers.^35,36^ Thus, identifying valuable nodes, including proteins listed above, in *MYC* mRNA translation for alternative Myc targeting, is imperative.

#### mTORC1

mTOR, a serine/threonine kinase, is the catalytic subunit of mTORC1 and mTORC2. mTORC1 shows a fundamental influence on protein synthesis. Under physiological conditions, mTORC1 phosphorylates 4EBP1 and thus frees the eIF4G binding pocket in eIF4E, thereby facilitating the initiation of mRNA translation. Certainly, Myc protein synthesis process is also coordinated by mTORC1. As pharmacological inhibition of mTORC1 by rapamycin on granulocytes has shown, the expression of endogenous *MYC* is downregulated, while the cellular levels of Myc mRNA is unaffected.^37^ Due to the compromised stability and solubility, rapamycin might not be eligible for clinical use to alternatively target Myc. MLN0128, a next-generation inhibitor of mTOR after rapamycin and its analogs, is extensively evaluated in clinical trials from phase 1 to phase 2.^38,39^ Phosphorylation of 4EBP1 by mTORC1, a poised status for the initiation of mRNA translation, is potently inhibited by MLN0128.^40^ More importantly, tumor burden of Eµ-Myc transgenic mouse model is reduced. Of note, mTORC1-dependent 4EBP1 phosphorylation is positively correlated with Myc overexpression in diffuse large B-cell lymphoma patients.^40^ Thus, potent mTORC1 inhibitors like MLN0128 could be valuable candidates for Myc-driven cancer treatment.

#### eIF4A

Directly targeting mTOR might fail to inhibit the translation of *MYC* mRNA. Due to the insufficient sequestration of eIF4E by endogenous levels of 4E-BPs plus independence of eIF4E of *MYC* mRNA translation in the case of internal ribosome entry segment (IRES) element in the 5′-UTR,^41^ druggable and meaningful nodes at downstream of mTORC1 become attractive. Unlike eIF4E, there is always a place for eIF4A helicase activity in *MYC* mRNA translation to address the structure impediment caused by G-quadruplexes. Silvestrol, an inhibitor of the eIF4A helicase, suppresses the Myc protein levels without influencing *MYC* mRNA levels in SW480 cells.^41^ It also inhibits tumor growth in a mouse model of colorectal tumorigenesis.^41^ Similarly, eFT226, a potent and selective eIF4A inhibitor, compromises the G-quadruplex-unwinding activity of eIF4A and interferes with the assembly of the eIF4F initiation complex.^42^ Therefore, eFT226 may have a promising future in treating tumors that harbor overexpression or amplification of oncogenes, such as *MYC*, *CCND1/3*, *BCL2*, or *MCL-1*, according to the preclinical efficacy studies across hematological tumor models.^42^ In addition, results of Phase 1–2 study of eFT226 in advanced solid tumor malignancies are encouraging (ClinicalTrials.gov: NCT04092673).

#### Destabilizing *MYC* mRNA

*MYC* mRNA stability is governed by mRNA polyadenylation (poly(A)) and deadenylation via a series of proteins. Pre-mRNA with cytoplasmic polyadenylation element (CPE), such as *MYC* mRNA, acquires a long poly(A) tail in the nucleus, which fades in the cytoplasm.^43^ The *MYC* mRNA could be recognized and bound with cytoplasmic polyadenylation element-binding protein (CPEB) through the CPEs of *MYC* mRNA. Furthermore, CPEB mobilizes Caf1 deadenylase and Tob to negatively regulate the expression of Myc by accelerating the deadenylation and decay of its mRNA.^43^ Intriguingly, the CPEB family of proteins is downregulated in human cancers.^44^ Thus, with its inhibition on *MYC* mRNA stability, approaches to reactivate the CPEB expression might be a promising approach for alternative targeting Myc.

Also, RNA-binding proteins such as coding region instability determinant-binding proteins (CRD-BPs) are key regulators in controlling mRNA decay rate and should be taken into account when *MYC* mRNA stability is involved.^45^ The coding region instability determinants (CRDs) are critical sequences for mRNA stability and critical binding sites for CRD-BPs.^45^ If a CRD-BP binds to *MYC* mRNA, then *MYC* mRNA would be granted immunity from endonucleolytic attack.^45^ Otherwise, CRDs of *MYC* mRNA would be exposed to endonuclease, resulting in degradation. Among numerous RNA-binding proteins, the IGF2BP-proteins (alternative names: CRD-BPs, IGF-II mRNA binding proteins, or zipcode binding proteins), participate in physiological processes, including regulation of localization, translation, or turnover of the target transcripts.^46^ IGF2BP1 stabilizes the mRNA of Myc together with four other proteins, including HNRNPU, SYNCRIP, YBX1, and DHX9.^46^ Importantly, IGF2BP1 and its homologs show an oncofetal expression declining towards the end of embryogenesis, while a barely detectable expression is noted in the postnatal stage, but rapid de novo synthesis occurs in various tumor.^46^ Hence, it would be a reasonable candidate to alternatively target Myc for cancer treatment. Typically, one structure-specific small molecule inhibitor of IGF2BP1, named BTYNB, destabilizes *MYC* mRNA and leads to a significant downregulation of Myc protein in melanoma cell line SK-MEL2.^47^

### Targeting Myc stability

Myc protein is tightly controlled by the ubiquitin–proteasome system (UPS) in normal tissues and has a relatively short half-life (around 20 min) under physiological conditions.^48,49^ However, it is frequently dysregulated in cancer cells via multiple mechanisms and mimics the phenotype associated with Myc overexpression. UPS, the main cellular digestion factory of proteins, is a highly specific mechanism powered by adenosine triphosphate (ATP). Proteins undergo two steps in the UPS process. First, the target protein is covalently tagged with poly-ubiquitin. Second, the tagged protein with both the correct number of ubiquitin and the proper type of linkages is recognized and accepted by 26S proteasome that further deubiquitinates, unfolds, and degrades the tagged protein into small peptide fragments.^50^ Three steps underlie the covalent conjugation of ubiquitin to the target protein. First, ubiquitin is activated by a ubiquitin-activating enzyme (E1), which finally yields an adenylated ubiquitin in an ATP-dependent manner. Second, adenylated ubiquitin is transferred to an E2 ubiquitin–conjugating enzyme. Third, E3 ubiquitin ligase binds to the target protein and facilitates the E2-catalyzed transfer of adenylated ubiquitin to the lysine (K) residue in the target substrate. Subsequently, additional ubiquitin molecules are transferred to lysine 48 (K48) in the previously added ubiquitin to form poly-ubiquitin chains. Generally, E3 ubiquitin ligase is responsible for substrate specificity in UPS.^51^

To date, the most extensively studied E3 ubiquitin ligase for Myc is SKP1-cullin-1-FBW7 E3 ubiquitin ligase complex (SCF^Fbw7^). In this sophisticated complex, Fbw7 is the pivotal component with substrate specificity. It has three isoforms distributed in different subcellular locations, namely Fbw7α, Fbw7β, and Fbw7γ. Fbw7α and Fbw7γ are involved in the regulation of Myc protein turnover. Fbw7 serves as a scaffold protein, bringing together E2 cdc34 and Myc, and facilitates the E2-dominated transfer of activated ubiquitin molecules to Myc. The fate of the Myc protein controlled by Fbw7 depends on the phosphorylation of Myc at two sites. Serine 62 phosphorylation (pS62) by ERK and/or CDKs stabilizes Myc, while threonine 58 phosphorylation (pT58) by GSK-3β triggers polyubiquitination by the E3 ligase Fbw7 and degradation by the proteasome.^52^ pS62 of Myc is a pre-requirement for its further phosphorylation on threonine 58. Until phosphorylation on threonine 58 is accomplished, protein phosphatase 2A (PP2A) dephosphorylates pS62 of Myc and leads to the recognition of pS58-Myc by E3 ubiquitin ligase SCF^Fbw7^ and pS58-Myc degradation by 26S proteasome. Such an elegant protein degradation process comprises many proteins, and these components may become suitable targets to destroy Myc oncoprotein.

#### Ubiquitin-specific protease 28

As a ubiquitin-specific protease, Usp28 binds to Myc and antagonizes the activity of Fbw7, thereby stabilizing Myc.^53,54^ Accordingly, depletion of USP28 could mimic the effect of MYC depletion in some cell lines.^55^ Usp28-mediated Myc stabilization is required for tumor cell proliferation and has shown clinical significance in non-small cell lung cancer, breast cancer, intestinal cancer, gliomas, and bladder cancer.^55^ Recently, [1,2,3] triazolo [4,5-d] pyrimidine derivatives have been reported as potent and selective cellular inhibitors of USP28. Among these derivatives, one compound not only showed high potency and selectivity on the inhibition of USP28 but also suppressed the colony formation, cell proliferation, cell cycle at S phase, migration, and the EMT process in gastric cancer cell lines.^56^

#### S-Phase kinase-associated protein 2

S-Phase kinase-associated protein 2 (Skp2), another ubiquitin ligase F-box protein identified for Myc but different from Fbw7, promotes Myc transcriptional activity through its E3 ubiquitin ligase activity, acting as a transcriptional coactivator. Intriguingly, Skp2 itself is a direct target gene of the transcription factor Myc as clarified by the fact that human myeloid leukemia K562 cells show Myc-induced Skp2 expression at both mRNA and protein levels.^57^ Skp2 is often overexpressed in various human cancer types and is also considered as an oncoprotein just like Myc. Meanwhile, Skp2 is a druggable oncoprotein, which makes it not only a primary target for Skp2-overexpressing cancer types but also a candidate for alternatively targeting Myc. Chan et al. reported a specific Skp2 inhibitor, SZL-P1–41, which selectively suppressed Skp2 E3 ligase activity and showed significant antitumor activities in multiple animal models.^58^

#### Inhibitors of PP2A

PP2A promotes Myc degradation, as mentioned earlier. Two endogenous inhibitors of PP2A, inhibitor-2 of PP2A (also known as SET oncoprotein) and cancerous inhibitor of PP2A (CIP2A), compromise PP2A as a negative regulator on Myc.^59^ These PP2A inhibitors are often overexpressed in several tumor types. For example, CIP2A is overexpressed in head and neck squamous cell carcinoma, colon cancer, and 39% of breast tumors, and this overexpression fashion of CIP2A is positively relevant to poor prognosis in multiple myelomas.^59,60^ SET is overexpressed in malignant brain tumors, tumors of the head and neck region, and testicular cancer.^59^ In acute myeloid leukemia, the overexpression of SET leads to poor outcomes by contributing to PP2A inhibition.^61^ Therefore, targeting SET or CIP2A may show great promise in destroying Myc. Indeed, an erlotinib derivative TD19 has been reported to inhibit SET and interrupt the association between SET and PP2A.^62^ Also, TD19 triggers cell apoptosis in triple-negative breast cancer cells and reduces tumor size and tumor weight, but not body weight in a xenograft model.^62^ Recently, a small molecule, DT1154, has been reported as an activator of PP2A, and it compromised the stability of Myc oncoprotein induced by HER2 signaling and suppressed tumor growth of a mouse model of HER2 amplified plus with Myc deregulated breast cancer.^63^ Therefore, it can be a favorable candidate for Myc-dysregulated cancer treatment.

#### Inhibitors of Polo-like kinase 1

Polo-like kinase 1 (PLK1) is an important component that contributes to the protein degradation of Myc oncoprotein. It is a serine-threonine kinase involved in mitosis and cytokinesis.^64^ PLK1 is found to be overexpressed in non-small cell lung cancer, head and neck cancer, esophageal cancer, gastric cancer, melanomas, breast cancer, ovarian cancer, endometrial cancer, colorectal cancer, gliomas, and thyroid cancer.^65^ Also, its overexpression is often related to the worse prognosis.^65^ Considering its essential role in cell cycle regulation, its close relevance to the mechanism of resistance to some chemotherapeutic drugs such as doxorubicin, paclitaxel, metformin, and gemcitabine,^66^ as well as its physiological function on Myc stability, it could be a qualified candidate for Myc-deregulated cancers. Glioma progression post-temozolomide treatment often confers Myc genomic amplification and/or pathway activation; PLK1 inhibition sheds light on this process.^67^ As recently reported, a PLK1 selective inhibitor, volasertib, showed a specific and significant inhibitory effect on Myc-overexpressing glioma cells.^67^ Likewise, PLK1 inhibition by volasertib impaired Myc expression in Diffuse large B-cell lymphoma (DLBCL) cell lines.^68^ Volasertib monotherapy displayed acceptable adverse events and favorable antileukemic activity in the phase 1 study of acute myeloid leukemia.^69^ The use of volasertib also achieved great success in combination with other inhibitors in clinical trials.^70,71^ These inspiring results, together with the negative affects of volasertib on prognosis, support the therapeutic potential of PLK1 inhibitors including volasertib.

#### PROTAC specific for Myc protein

As mentioned above, PROTACs are an emerging modality of anti-tumor drug. And the major advantage of it is a long-sustained and specific degradation of target proteins. They are heterobifunctional molecules with a ligand for binding target, another ligand that recruits an E3 ubiquitin ligase, and a linker connecting these ligands. Thus, a PROTAC agent could hijack the E3 ubiquitin ligase for degrading the specific oncoproteins that recognized by its ligand, potently and constantly. Over a hundred PROTACs have been reported for degrading proteins of interest and chemical biology, and drug development.^72^ In the case of targeting Myc oncoprotein, an obvious obstacle is lack of high-affinity ligands to bind and trap Myc. So, the major priority is to find some qualified small molecules which does not even need to show pharmacological inhibition of Myc oncoprotein. Besides, screening molecules that could bind or interfere with Myc–Max would also be helpful to develop related PROTACs for Myc oncoprotein since either degrading Myc or Max could both lead to the inhibition of Myc downstream pathway.

### Targeting the Myc–Max heterodimer

As a transcription factor, Myc generally accomplishes its physiological functions through HLH-Zip domain-based Myc–Max heterodimerization.^73^ The heterodimer complex of Myc–Max binds specific motifs of corresponding target genes, such as E-box motif CACGTG, through the BR/HLH/LZ domain.^73^ After binding to the correct site, the Myc–Max heterodimer recruits other necessary factors to modulate the chromatin structure. For example, Myc collaborated with transformation/transcription domain-associated protein (TRRAP), which is a component of a large complex having histone acetyltransferase (HAT) activity. Specifically, Myc first binds to the promoter region. Then, Myc recruits TRRAP and promotes the preferential acetylation of histone H4 at a single nucleosome. The acetylation of histone H4 alters the chromatin structure and facilitates efficient accessibility and binding of Myc–Max transcriptional-activator complexes to genomic targeted DNA sequences. This heterodimer complex of Myc–Max activates the transcription of diverse genes. Further, the protein products of these genes finally participate in multiple fundamental processes.

Considering the dimerization of Myc and Max is indispensable for Myc to govern diverse groups of genes, disassociating the Myc–Max heterodimer complex can be a conceivable and potent approach to abrogate the oncogenic influences incurred by Myc dysregulation directly. Alina Castell et al. used bimolecular fluorescence complementation to screen reliable and effective Myc–Max inhibitors and reported that a compound, MYCMI-6, bound to the Myc BR/HLH/LZ domain directly and selectively with high affinity and interfered with the interaction between Myc and Max, but not affected the protein level of Myc.^74^ Importantly, MYCMI-6 administration reduced cell growth and survival in cancer cell lines harboring a high Myc protein level but was not cytotoxic to normal human cells. Likewise, precluding the heterodimer formation by stabilizing the Max homodimer would also be an alternative way to target dysregulated Myc for cancer treatment. As Struntz et al. reported recently, a small molecule named KI-MS2-008 induced the homodimerization of Max–Max and further led to global changes both in vitro and in vivo at the transcriptome level, which was similar to Myc inactivation.^75^

Another feasible molecule to destroy the Myc–Max heterodimer would be the dominant allele of Myc, Omomyc. Soucek et al. first propelled the design of a dominant-negative mutant of Myc as a tool to investigate the outcomes of systematically inhibiting Myc in transgenic mouse models.^16^ Omomyc relieved the concerns about the catastrophic side effects of the inhibition of Myc on normal cells. As a Myc-derived peptide, Omomyc contains the bHLH-Zip domain of Myc but with four mutations (E63T, E70I, R77Q, and R78N). Hence, Omomyc binds to Myc through its bHLH-Zip domain and prevents the interaction between Myc and Max. Hijacked Myc would lose its control on downstream target genes. Despite the enormous therapeutic potential of Omomyc for cancer treatment, most recent studies on Omomyc were based on retroviral vectors or transgenic models and thus not suitable for clinical translation. Meanwhile, Omomyc, on its own, demonstrated relatively poor delivery across physiological obstacles to the desired cellular compartment. Therefore, despite the great promise for cancer treatment, the therapeutic utility of Omomyc has been largely compromised due to the shortage of tumor cell penetration in vivo. Nevertheless, some modified Omomyc surfaced out and showed favorable outcomes. Omomyc linked with an N-terminal functional penetrating phylomer, namely FPPa-OmoMYC, showed a potent and unprecedented inhibitory effect on Myc oncoprotein.^76^ The anti-proliferative and pro-apoptotic pathways were activated specifically by FPPa-OmoMYC both in vitro and in vivo in triple-negative breast cancer cells. Hence, destabilizing the Myc–Max heterodimer by either destroying the heterodimer or occupying the binding interface between Max and Myc would be a promising way to target Myc alternatively for cancer treatment.

### Targeting accessibility of Myc to downstream genes

As mentioned earlier, Myc, as an important transcription factor, binds to the E-box element through its BR/HLH/LZ motif and regulates the elaborate downstream gene repertoire with diverse biological functions. The Myc–Max heterodimer is vital for the oncogenic activity of Myc. However, correct spatial localization and proper chromatin structure are highly important for the efficient access of Myc to its highly condensed target genes. Such efficient free access of Myc to downstream target genes is invariably necessary for the oncogenic activity of Myc. Collaborations are needed to alter the unfavorable chromatin structure and facilitate the binding of the Myc–Max transcription factor complex to target genes for the initiation of gene transcription.

Specifically, one prerequisite factor underlying the efficient access of Myc to target genes is nuclear pore localization. The localization of Myc in the nuclear pore is a prerequisite for its oncogenic activity. A recent study reported that the PIN1-mediated isomerization of Myc oncoprotein regulates its spatial distribution in the nucleus.^77^ PIN1, a phosphorylation-dependent prolyl isomerase, could recognize the Phospho-Ser/Thr-Pro motif and catalyze the proline-63 of serine-62 phosphorylated Myc oncoprotein, leading to the nuclear pore localization of serine-62 phosphorylated Myc. The spatial access of Myc oncoprotein to the inner basket of the nuclear pore promotes the recruitment of histone acetyltransferase GCN5 to bind and regulate the acetylation of local genes, which leads to expression of Myc target genes. Thus, oncogenic Myc triggers the transcription of downstream genes responsive to mitogen stimulation and activates proliferation and migration pathways. Given the contribution of PIN1 to the oncogenic activity of Myc, pharmacological silencing PIN1 may be a rewarding direction to go. Recently, Dubiella et al. reported a highly selective covalent inhibitor of PIN1 named Sulfopin.^78^ Sulfopin potently binds and inhibits PIN1, which induces downregulation of c-Myc target genes. And it also suppresses tumor initiation and tumor progression in murine and zebrafish models. Intriguingly, PIN1 is also found to facilitate PP2A-mediated destabilization of Myc oncoprotein.^79^ PIN1 interacts with Myc through its WW domain, which could recognize phosphorylated residues. Threonine-58 phosphorylation of Myc oncoprotein is necessary for its efficient trapping by Pin1. A *cis*-to-*trans* isomer change in PIN1-catalyzed Myc made Myc an ideal substrate of PP2A. Farrell and colleagues reported that PIN1 facilitated Myc–DNA binding and promoted the release of Myc and further degradation.^80^ Therefore, PIN1, as a double-functional interactor of Myc, is a valuable and promising target in the regulatory network of Myc pathway.^81^ Another factor looming in the background is epigenetic markers such as histone H3 K4/K79 methylation and H3 acetylation. Histone H3K4 methylation functionally regulates chromatin transcription and is involved in the Myc target gene expression repertoire. Recently, histone H3K4 methylation was reported to be hijacked by Myc oncoprotein in *Myc*-driven cancer. Hijacked H3K4 methylation offers convenient access for Myc oncoprotein to its genomic target genes, thus collaborating the oncogenic program for sufficient tumorigenesis in various cancer types driven by deregulated Myc. Myc oncoprotein rearranges signal modules inside tumor cells to augment its oncogenic power and these rewired signal components could, in return, become the vulnerability of cells harbored oncogenic Myc. As a common core subunit of one major H3K4 methylation enzyme in mammals, Dpy30 is a vulnerable node for Myc-driven lymphoma. Dpy30 directly regulates chromosomal H3K4 trimethylation on a genome-wide scale.^82^ Furthermore, it is often co-expressed with Myc across diverse cancer types in human. Dpy30 is significantly upregulated in human Burkitt lymphoma (BL) samples in comparison with non-BL subtypes. Both data sets from the *MYC*-driven lymphomagenesis mouse model and further analyses of a wide variety of cancer types indicate the strong association between Myc and Dpy30 overexpression without the limit of tumor types. Studies on cell lines and further analyses of microarray results have confirmed the importance of Dpy30 for the expression of Myc and the efficient access of Myc to target genes. Dpy30 heterozygosity significantly suppresses *MYC*-driven lymphomagenesis but does not affect the normal physiology, including lifespan. Because cells fail to combat oncogene-triggered apoptosis. Therefore, Dpy30 is vulnerable and a potential drug target for hyperactivated Myc oncoprotein. Considering its currently limited inhibitors including ASH2L-derived peptides, the question is how to find eligible inhibitors to target Dpy30.^83,84^ Similarly, another core subunit of chromatin remodeler, namely BPTF, is also a necessary component for the activation of the complete Myc transcription activity in fibroblasts.^85^ Moreover, BPTF expression positively correlates with *MYC*-induced transcriptome signatures in multiple cancer types in humans, such as BL, colorectal, prostate, and pancreatic carcinomas.^85^ The depletion of *Bptf* delayed tumor development and prolonged survival in pre-neoplastic pancreatic acinar cells.^85^ The pharmacological inhibition of BPTF with C620-0696, a potent inhibitor of BPTF, suppresses the expression of *MYC* in non-small cell lung cancer cells.^86^ Collectively, restraining the free access of Myc to downstream target genes can be another promising approach to treat Myc-driven cancer types.

### Targeting Myc with synthetic lethality

The alternative ways mentioned earlier to target Myc oncoprotein may confer some harmful effects to normal cells, due to lack of selectivity. Destroying critical nodes beneath the process of *MYC* transcription and *MYC* mRNA translation indiscriminately locks and inhibits the expression of other proteins that share the same expression pattern with Myc. For example, no inhibitor to date selectively inhibits BRD4 and spares the other members of the BET protein family while simultaneously downregulating the expression of oncogenic Myc.^87^ Given the nonredundant function of these BET proteins, less selective inhibition of BET proteins incurs severely adverse effects, which are unpredictable and unavoidable in this case.^87^ While targeting the translation process to alternatively target Myc, a similar dilemma also confronts. In terms of targeting regulatory components of Myc stabilization, disassociating the Myc–Max heterodimer or blocking the free access of Myc to downstream target genes, adverse effects on normal cells may also exist. Therefore, the therapeutic index of these inhibitors is important for the treatment of Myc-dysregulated cancers. That is, the optimal efficiency of a given inhibitor in cancerous cells and its minor toxicity in innocent normal cells are crucial.

Synthetic lethality could achieve the goal of alternatively targeting oncogenic Myc while nearly leaving normal cells untouched. Synthetic lethality was first stated by Wright and Dobzhansky in the context of fruit fly research and its concept is gradually applied to the cancer research area.^88,89^ The fundamental principle of synthetic lethality is that the combination of two gene mutations occurring either simultaneously or sequentially leads to death, while the adverse effect of single mutation of such a gene pair is buffered by another unmutated gene. From the molecular aspect, synthetic lethality is, essentially, a case of non-oncogene addiction and a vulnerability imparted by tumorigenesis. Targeting components synthetically lethal with Myc oncoprotein has catastrophic effects on Myc-overexpressing cancer cells without impacting the normal cells. As Myc oncoprotein is often overexpressed in ~70% of various human cancers, problem is to find the genes and their protein products that are dependent by Myc deregulated cells from diverse cancer types. At least, from the theoretical aspect, strategies based on synthetic lethality can be a more selective and potent way to alternatively target Myc oncoprotein for cancer treatment. Actually, around two decades ago, Hartwell and colleagues proposed that synthetic lethality could be used to identify new anticancer drug targets,^90^ especially targets that are synthetically lethal to known driver mutations. Around a decade later, the use of PARP inhibitor olaparib in patients carrying mutations of suppressor genes *BRCA1* and *BRCA2* demonstrated an obvious clinical benefit with less severe side effects, compared with the conventional chemotherapy, and propelled the clinical practice of synthetic lethality.^91^ Moreover, synthetic lethality may hold the promise of dealing with drug resistance against first-line therapy, which is a serious concern for cancer treatment. It is a reprogrammed signaling pathway acquired from artificial selection. And just like the natural tumorigenesis process, this reprogrammed signaling pathway confers relative adaptability but also imparts new vulnerability to cancer cells. Thus, synthetic lethality reshapes the future of alternatively and selectively targeting Myc oncoprotein for cancer treatment, despite of lacking clinically permitted effective small molecule that could treat *MYC*-driven cancer types based on synthetic lethality.

**Fig. 4:**
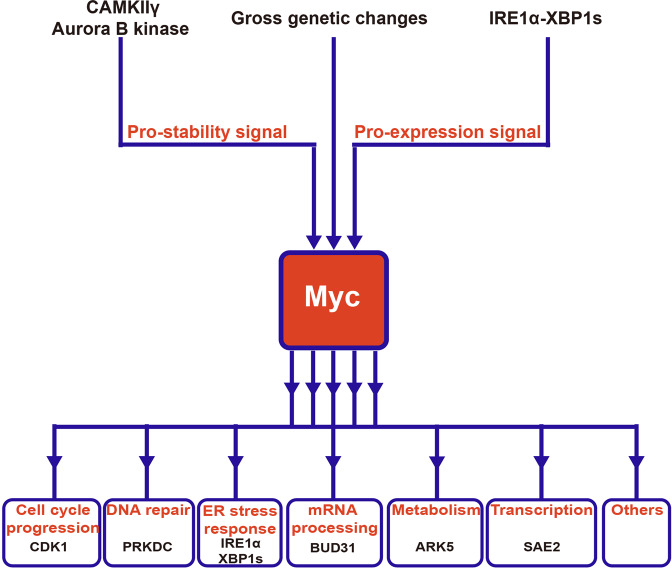
Rewired signal pathways by deregulated Myc in cancer and its related components available for synthetic lethality. Input signals, including pro-stability signals from CAMKIIγ or Aurora B kinase, pro-expression signals from IRE1α–XBP1s pathway, and gross genetic changes of Myc, lead to the deregulation of Myc. Multiple pathways, including cell cycle progression, DNA repair, ER stress response, mRNA processing, metabolism, and transcription, were rewired by the output signal from oncogenic Myc to meet its oncogenic needs. Among these Myc-educated signal pathways, some related components indicated here have been verified as synthetic lethal partners of oncogenic Myc

As high-throughput screening technology continues to thrive, more than a hundred candidate genes, which might be potentially synthetically lethal to Myc-dysregulated oncoprotein, has successfully identified. It not only depicts the landscape of rewired and also addicted signal pathways by deregulated Myc, but also offers some vulnerable targets to selectively kill cells harbored hyperactivated Myc. Some of those pathways and related components are shown in Fig. 4. Definitely, diverse genes synthetically lethal to dysregulated Myc should be extensively investigated and assessed. Reliable synthetically lethal combinations are exceedingly important for drug development. Here, some meaningful preclinical studies were introduced to share the recent progresses in this field.

#### Myc overexpression and ER stress inhibition

Increased demand for energy is one of the emerging hallmarks of cancer. Dysregulated Myc enhances ribosome biogenesis and protein synthesis, which are energy-intensive bioprocesses. Unfolded protein response (UPR), as a cellular homeostatic program activated by an excess of unfolded or misfolded proteins in the endoplasmic reticulum (ER) lumen, is thus engaged in Myc overexpression. Hence, the relative dependence of Myc-driven cancer on the UPR system may impart unique vulnerability and be a feasible target to selectively kill cancer cells characterized by Myc overexpression while sparing the normal ones. Three major stress sensors are critical for the execution of elaborate UPR. For instance, inositol-requiring enzyme 1 (IRE1) comprises an ER-luminal sensor domain. This domain recognizes unfolded proteins and cytosolic kinase and endoribonuclease domains that mediate responses through downstream effectors. Two paralogs, IRE1α and IRE1β, exist in mammalian cells. IRE1α is universally expressed, while IRE1β is restricted to the epithelium of the gastrointestinal tract. Inhibiting the endoribonuclease (RNase) activity of IRE1, especially IRE1α, can lead to synthetic lethality in the case of Myc overexpression. ER stress threatens the cell hemostatic environment, and then IRE1 is activated through dimerization and autophosphorylation. Activated IRE1 removes 26 nucleotides from unspliced X-box-binding protein 1 (*XBP1u*) mRNA to generate spliced *XBP1* (*XBP1s*) through its RNase activity. The protein product of *XBP1s* is a transcription factor involved in the expression of *MYC* gene and numerous genes responsible for maintaining ER homeostasis.^92^

Myc activates the pro-survival IRE1α/XBP1 pathway in a wide range of human and murine cancer types, including BL, chronic lymphocytic leukemia, and hepatocellular carcinoma. A highly specific IRE1α RNase inhibitor B-I09 decreases the protein level of XBP1s in a dose-dependent manner in lymphoma cell line P493 cells with a high Myc protein level in the case of ER stress.^93^ Also, B-I09 compromises the cell proliferation and viability of P493 cells with high-Myc-expression in a dose-dependent manner, while the effects of B-I09 are modest in low-Myc and no-Myc cells. Moreover, apoptosis of Myc-overexpressed cells induced by B-I09 is much more severe than that induced by doxorubicin, a traditional chemotherapeutic drug targeting highly proliferating cells, or that induced by JQ1. B-I09 also sensitizes the effect of standard therapies currently used to treat BL clinically in BL cells. Similarly, Na Zhao and Xi Chen also revealed the synthetic lethal interaction between Myc and IRE1 in the context of breast cancer.^94^ They discovered a pharmacological inhibitor of IRE1, namely 8866. The same antitumor effect was also displayed through the inhibition of IRE1. The inhibitor 8866 could selectively restrain the Myc-overexpressing tumor growth in preclinical patient-derived human breast cancer xenograft models and genetically engineered mouse models with efficacy comparable to that of the standard-of-care chemotherapy docetaxel. Hence, alternatively targeting Myc oncoprotein through the inhibition of IRE1α could be a promising approach for cancer treatment. Highly specific inhibitors of IRE1α combined with current first-line drugs, may also be a favorable choice for treating Myc-dysregulated cancer types.

#### Myc overexpression and cyclin-dependent protein kinase 1 inhibition

The pro‐oncogenic activity of Myc is partly characterized by the potent induction of cell proliferation by promoting G1- to S-phase transition during cell cycle progression.^95^ In fact, reinforced Myc expression in quiescent cells is sufficient to promote cell cycle entry.^95^ The enforced control of the cell cycle by Myc oncoprotein relies much more on the participation of specific proteins. Such dependence of specific proteins may also make them vulnerable targets for dealing with “undruggable” Myc oncoprotein. Among these, cyclin-dependent protein kinase 1 (CDK1) can be a feasible target to selectively cause the collapse of cancer cells with aberrant Myc expression. CDK1, one of the highly conserved serine/threonine protein kinases, is a catalytic subunit of the M-phase-promoting factor. Cyclin-dependent protein kinases (CDKs), together with their regulatory partner cyclin, govern cell cycle progression. Myc oncoprotein induces transformation basically through cell cycle stimulation.^95^ Three leading mechanisms are required for cell cycle stimulation by Myc: upregulation of CDKs and cyclins, downregulation of CDK inhibitors p15 and p21, and degradation of p27.^95^ The phosphorylation of Thr-187 in p27 is a prerequisite for its degradation by the ubiquitin–proteasome system. CDK1 and CDK2 both phosphorylate the critical phosphorylation site of p27. While CDK2 is absent, the phosphorylation is mainly accomplished by CDK1.^95^ The synthetic lethality between CDK1 and Myc has been reported previously.^96,97^ Using small interfering RNA (siRNA), CDK1 has been discovered as a potential synthetic lethal partner of Myc oncoprotein. Further inhibition of CDK1 using either RNAi or small-molecule inhibitors justifies its synthetic lethal ability in Myc-dependent cells. Moreover, a small molecule purvalanol A specifically inhibits CDK1 and selectively induces apoptosis in Myc-overexpressing cells.^98^ Also, purvalanol A significantly decreased tumor growth of Myc-dependent lymphoma and hepatoblastoma mouse models.

#### Myc overexpression and inhibition of Ca^2+^/calmodulin-dependent protein kinase II γ

Despite a broad range of proteins capable of regulating the stability of Myc oncoprotein, not all of these regulators are specifically responsible for the regulation of Myc oncoprotein stability. Pharmacologically targeting a universal regulator of protein stability may indiscriminately lead to damage to multiple proteins and unselectively kill normal cells. Therefore, an appropriate component should be carefully chosen when focusing on the alternative approaches to target Myc oncoprotein. Recently, CAMKIIγ was recognised as a direct stabilizer of Myc oncoprotein. Ca^2+^/Calmodulin-dependent protein kinase II γ (CAMKIIγ) is a serine/threonine kinase. The potential synthetically lethal interaction of CAMKIIγ with aberrant Myc oncoprotein has been recognized in synthetic lethal screening performed by Masafumi Toyoshima and co-workers. They identified 102 potential synthetically lethal interactions with c-Myc overexpression in a collection of ∼3300 druggable genomes via high-throughput siRNA screening.^99^ Enlightened by their brilliant work, the synthetic lethality between Myc overexpression and inhibition of CAMKIIγ in T-cell lymphoma was confirmed, and the novel role of CAMKIIγ in the regulation of Myc stability was reported as the molecular mechanism.^100^ As the principal degradation marker of Myc, Serine-62 phosphorylation (pS62) by kinases such as ERK and/or CDKs stabilizes Myc while threonine-58 phosphorylation (pT58) by GSK-3β leads to polyubiquitination by E3 ligase Fbw7 and further degradation by the proteasome. CAMKIIγ directly phosphorylates Myc protein at serine-62, leading to further stabilization of Myc. The stabilization of Myc transcription factor by CAMKIIγ further leads to the collapse of normal tight control of the protein level of Myc. Dysregulated Myc ultimately propels the tumorigenesis. More important, CAMKIIγ is not only critical for the maintenance of T-cell lymphoma that characterised by Myc deregulation but also frequently overexpressed in T-cell lymphoma patients. A specific pharmacological inhibitor of CAMKIIγ berbamine destabilizes Myc oncoprotein and efficiently suppresses T-cell lymphoma in mouse models. These findings indicated that inhibiting CAMKIIγ can be a more potent and selective way to alternatively target Myc for cancer treatment.

#### Myc overexpression and inhibition of Aurora B kinase

Aurora B kinase is a serine/threonine kinase that is vital for its integral role in the regulation of cell division, including spindle checkpoint, chromosome segregation, and cytokinesis. It is the catalytic subunit of the chromosomal passenger complex, which is required for mitotic progression and cytokinesis.^101^ Aurora B kinase along with inner centromeric protein and survivin localizes to centromeres and spindle midzone during metaphase to anaphase transition.^101^ Overexpression of Aurora B kinase has been reported in a broad spectrum of cancers and is related to poor overall survival.^102^ Intriguingly, Aurora B kinase is the final protein product of a downstream target of Myc transcription factor, and is considered essential for the maintenance of malignant state in Myc-driven cancer.^101^ Such implications make Aurora B kinase a rational drug target, especially in Myc overexpressed cancers. Additionally, the synthetic lethal interaction between Myc overexpression and inhibition of Aurora B kinase has been reported to act as a therapeutic strategy for killing tumor cells that overexpress Myc.^103^ VX-680 (MK-0457) is an aurora kinase inhibitor that selectively kills cells with high Myc protein levels. Such selective killing comes from prompt apoptosis and delayed autophagy, showing therapeutic efficacy in an animal model of lymphoma.^103^ Unfortunately, clinical trials of VX-680 showed limited positive feedback, which may have multiple reasons and need further reflection.^104^ But methods that alternatively target Myc through inhibition of Aurora B kinase still hold promising therapeutic value. Recently, another aspect on the interaction between Aurora B kinase and Myc has been observed. Just like CAMKIIγ, Aurora B kinase also directly phosphorylates Myc at serine 62, leading to stabilization of Myc and further oncogenic activity of Myc. Also, AZD1152 is a highly potent and selective inhibitor of Aurora B, and is shown to have anti-tumor efficacy and tolerable toxicity in a preclinical setting of relapsed/refractory diffuse large B‐cell lymphoma and acute myeloid leukemia.^105–107^

#### Myc overexpression and BUD31

Deregulated Myc is found to be accumulated inside cells, leading to intensive transcription of target genes. Increased amount of pre-mRNA of downstream genes governed by hyperactivated Myc make the spliceosome overloaded, causing unique vulnerabilities in Myc-dependent cells. Indeed, the core subunit of spliceosome BUD31 is required to tolerate the overload of pre-mRNA due to dysregulated Myc. Also, a genome-wide screening of Myc-synthetic lethal genes in human mammary epithelial cells revealed potential lethal combination between Myc overexpression and *BUD31*.^108^ Further cell line works showed that knockdown of BUD31 significantly and selectively inhibited the proliferation of *MYC*-driven breast cancer cells. Moreover, loss of BUD31 inhibited tumor growth and metastatic expansion in Myc-dependent triple-negative breast cancer mouse model. Pharmacological inhibition of BUD31-involved spliceosome complex significantly suppressed colony formation, induced apoptosis in an Myc-selective manner, and showed a favorable anti-tumor effect as well as impaired metastatic potential.

Several other studies have reported further meaningful synthetic lethal interactions related to Myc overexpression. A pooled kinase shRNA library screening and next-generation deep sequencing have revealed that inhibition of a protein kinase DNA-PKcs, which is involved in non-homologous end-joining DNA repair, using RNAi (RNA interference) preferentially killed Myc-overexpressing human lung fibroblasts.^109^ Unfortunately, selective and potent drug-like small molecule inhibitors of DNA-PKcs are lacking for a long time. However, Fok et al. recently reported a potent, as well as selective, DNA-PKcs inhibitor named AZD7648.^110^ This novel inhibitor is being evaluated as either monotherapy or in combination with doxorubicin or olaparib in a Phase I/II first-in-human study. Collectively, it may hold promise for the future trials in Myc-driven cancers. The vulnerability exploited here is partly due to DNA-PKcs dependent modulation of Myc expression, and DNA-PKcs involved repair of DNA damage that resulted from Myc. In fact, Myc also transcriptionally orchestrates the genes involved in homologous DNA recombination and thus, confers some vulnerable nodes to cells that harbor both high levels of Myc oncoprotein and homologous DNA recombination defect. As reported by Andrei Goga and colleagues, Myc oncoprotein is overexpressed in TNBC, resulting in increased activity of Myc pathway.^98^ Overactivation of Myc pathway in TNBC leads to synthetic lethal outcomes with CDK inhibition. Similar synthetic lethal interaction has also been reported later by Jason P.W. Carey and his colleagues. They discovered an intriguing phenomenon that the PARP inhibitors when combined with Myc inhibition leads to lethality in triple-negative breast cancers (TNBCs).^111^ And this specific synthetic lethal interaction is independent of BRCA status. Through drug combination of cyclin-dependent kinase inhibitor dinaciclib, which downregulates Myc expression, and PARP inhibitor niraparib, the DNA damage is increased and the homologous recombination in TNBC cell lines is downregulated.^111,112^ Further combined administration of dinaciclib and PARP inhibitors such as olaparib and velaparib exhibited similar synergism, leading to subsequent downregulation of epithelial-mesenchymal transition (EMT) and cancer stem-like cell phenotypes. Considering the acquired drug resistance by breast cancer patients from clinical monotherapy treatment of niraparib or other PARP inhibitors and together with the frequent presence of Myc overexpression in TNBC patients, finding a candidate drug based on the synthetic lethality conferred by Myc oncoprotein has become quite promising. Indeed, drug resistance is very common throughout the cancer treatment. A broad range of approaches could assist in precisely tackling these difficulties. Perhaps, the newly acquired dependences or rather addictions after administration of first-line drug also left a loophole for alternative selective killing of these dysregulated cells through synthetic lethality.

## Peer into the future: a hint from omomyc

Although undruggable Myc lacks traditional binding pockets for small molecules, the success of Omomyc prompts us peer into the future of Myc targeting. To some extent, Omomyc or its derives could be counted as antibody-like agents for their binding pockets, or rather, arms. That is why most concerns in the early days are commonly voiced for the inaccessibility of Omomyc to nuclear compartment. Actually, it is a fundamental dilemma met by antibody drugs to target cytoplasm proteins or even nuclear proteins like Myc.

However, Omomyc overcame such dilemma and made great progress in a preclinical stage. The mechanisms underlying the efficient uptake of Omomyc deserve further investigation. The influx of purified Omomyc could be attributed to its protein transduction domains or cell-penetrating peptides. But the mechanism behind this may lie largely in macropinocytosis, which is a non-receptor dependent pathway for nutrient scavenging and is first coined in the background of *RAS* mutant glioblastoma cells.^113^ Either the genomic backgrounds of cell lines and mouse models or the serum-free treatment in the viability test of Omomyc would inevitably lead to an intensified macropinocytosis. The NCSLC cell lines (H1299, H1975, and A549) used to justify the uptake of Omomyc harbor *NRAS*,^114^ *EGFR*,^115^ and *KRAS*^116^ mutation, respectively. These mutations could trigger downstream actin remodeling for macropinocytosis initiation and enhance macropinocytosis in diverse cell lines.^113,117,118^ As the *KRAS* mutant lung adenocarcinoma mouse model has an active macropinocytosis due to intensified KRAS signal, Omomyc was predominately located in the lung tumors as Laura Soucek and colleagues recently showed.^115^ Serum starvation has also been reported as an inducer to augment inherent macropinocytosis and recommended as a preferred procedure in the assay of determining macropinocytic index.^119,120^

macropinocytosis is usually used for nutrient-scavenging and may help direct high molecular weight agents to their destinations under some circumstances. As a test shows, uptake of linear peptides, peptide macrocycles, stabilized helices, β-hairpin peptides, and cross-linked helix dimers by cancer cells is positively correlates to their macropinocytosis levels.^121^ Generally, cancer cells have demanding nutrient needs to thrive and exhibit high inherent macropinocytosis levels, especially *KRAS* mutant cells. Thus, it may offer an opportunity to overcome the barrier and deliver structurally-modified peptides or antibody agents to intracellular compartments to target Myc in the circumstances of high basal macropinocytosis level. However, proper peptides or antibody-based agents are limited so far. Besides Omomyc-based peptides, one current promising agent is a fusion protein of a U box-type ubiquitin ligase (E3) and Max, designated Max-U. Taken together, macropinocytosis does hold some promise for utilizing as peptides and antibody delivery route, but further study exploiting this pathway is still needed.

## Conclusion

As a long-pursued target, Myc is still considered undruggable directly by small molecules. The main approaches to alternatively target Myc have been summarized here into to 6 principles: (a) targeting the process of *MYC* gene transcription; (b) inhibiting *MYC* mRNA translation; (c) destructing the stability of Myc oncoprotein; (d) abrogating the dimerization of Myc–Max; (e) blocking the access of Myc to genomic targets; and (f) selectively killing Myc-dependent cells with synthetic lethality. Moreover, small molecules or peptides that aim at these alternative nodes have shown some progression as listed in Table 1. The approaches mentioned above also offered a paradigm for other “undruggable” oncoproteins such as Ras. When formulating feasible approaches that alternatively target Myc oncoprotein, one important consideration is that priority should be given to the diverse molecular backgrounds that support the tumorigenesis and maintenance of Myc-deregulated cancers. Cancer treatment depending on such molecular characters might popularize in the near future. In fact, targeting Myc oncoprotein through synthetic lethality with other druggable proteins fits this concept well. Such approaches could selectively kill cells harboring high-levels of Myc oncoprotein while leave the normal cells untouched, and this theoretically outperforms the methods based on the other 5 principles. Although there is no clinically approved drugs or small molecules available so far to confer such benefit to clinical practice, hope has rooted in the successful utilization of PARP inhibitors in *BRCA1/2* mutant breast cancers. Likewise, despite Omomyc has not engaged in clincal stage, its preclinical progresses have justified its potency and help us peer into the promising future of peptide or antibody delivery through macropinocytosis route.

**Table 1:** Alternative nodes to target Myc through small molecules or peptides

Target	Compound	Status	References
*Myc* transcription			
G-quadruplex	QN-1	Preclinical	^22^
	APTO-253	Phase 1	^23^
BRD4	AZD5153	Phase 1	^29^
	GSK525762	Phase 1–2	^27^
	dBET1	Preclinical	^31^
*Myc mRNA translation*			
mTORC1	MLN0128	Phase 1–2	^38,39^
eIF4A	Silvestrol	Preclinical	^41^
	eFT226	Phase 1–2	^42^
IGF2BP1	BTYNB	Preclinical	^47^
*MYC stability*			
USP28	[1,2,3] triazolo [4,5-d] pyrimidine derivatives	Preclinical	^56^
Skp2	SZL-P1–41	Preclinical	^57,58^
SET	TD19	Preclinical	^62^
PLK1	Volasertib	Phase 3	^69–71^
PROTAC	Not availabe		
*Myc–Max heterodimer*			
Myc–Max	MYCMI-6	Preclinical	^74^
	KI-MS2-008	Preclinical	^75^
	Omomyc	Preclinical	^16^
	FPPa-OmoMYC	Preclinical	^76^
*Accessibility of Myc to downstream genes*			
PIN1	Sulfopin	Preclinical	^78^
Dpy30	ASH2L-derived peptides	Preclinical	^83,84^
BPTF	C620-0696	Preclinical	^86^
*Synthetic lethality*			
IRE1α	B-I09	Preclinical	^93^
	8866	Preclinical	^94^
CDK1	Purvalanol A	Preclinical	^98^
CAMKII γ	Berbamine	Preclinical	^100^
Aurora B kinase	VX-680 (MK-0457)	Phase 2	^104^
	AZD1152	Phase 1	^105–107^
BUD31	Not available		
DNA-PKcs	AZD7648	Phase 1–2	^110^
CDK	Dinaciclib	Phase 3	^112^
